# Interpersonal emotion regulation and family functioning in the relationship between anhedonia and depressive symptoms in adolescents: a moderated mediation model

**DOI:** 10.3389/fpsyg.2026.1863238

**Published:** 2026-10-29

**Authors:** Qiwen Tang, Siying Lin, Fanggui Tang

**Affiliations:** 1 Institute for Advanced Studies in Education, Faculty of Education, East China Normal University, Shanghai, China; 2 Changsha Foreign Languages School, Changsha, China; 3 College of Education Science, Hengyang Normal University, Hengyang, China

**Keywords:** adolescents, anhedonia, depressive symptoms, interpersonal emotion regulation, family functioning

## Abstract

A questionnaire survey was conducted with 1,635 students from two secondary schools in Hunan Province to examine the statistical roles of interpersonal emotion regulation and family functioning in the relationship between anhedonia and depressive symptoms in adolescents. The results showed that (1) after controlling for grade and gender, anhedonia was significantly and positively associated with depressive symptoms; (2) a continuous-variable model based on the full sample indicated a significant partial mediating effect of interpersonal emotion regulation on the association between anhedonia and depressive symptoms; and (3) family functioning moderated the association between anhedonia and interpersonal emotion regulation, such that lower family functioning scores (i.e., healthier family functioning) were associated with stronger conditional indirect effects linking anhedonia to depressive symptoms through interpersonal emotion regulation. When BDI-II-C Items 4 (loss of pleasure) and 12 (loss of interest) were removed from the depressive symptom total score and the analyses were repeated, the main results remained stable. An exploratory sensitivity analysis using the BDI-II-C cutoff revealed a significant mediating pathway in the below-cutoff group, whereas the regression association between interpersonal emotion regulation and depressive symptoms was not significant in the at-or-above-cutoff group. Given that grouping by the cutoff of the same dependent variable narrows the within-group score range and may affect within-group regression estimation, these subgroup results are reported only as exploratory sensitivity analyses. These findings suggest that interpersonal emotion regulation and family functioning are important factors in understanding the statistical association between anhedonia and depressive symptoms in adolescents.

## 1 Introduction

Adolescence is a critical period of transition from childhood to adulthood and a stage of rapid social, emotional and cognitive development. Rates of depression rise markedly after the onset of puberty, and depression is associated with persistent impairment in social functioning and with elevated suicide risk (Thapar et al., 2022). The World Health Organization has noted that depression and anxiety are among the leading causes of illness and disability among adolescents and that suicide is the third leading cause of death among those aged 15–29 years (World Health Organization, 2024). A meta-analysis of Chinese studies conducted between 2015 and 2024 likewise indicates that the detection rate of depressive symptoms among children and adolescents remains relatively high (Feng and Wang, 2025). Examining the correlates of adolescent depressive symptoms and their statistical associations, therefore, carries both theoretical and practical significance.

Anhedonia, one of the core clinical symptoms of depression (American Psychiatric Association, 2022) and a transdiagnostic feature of considerable research value (He et al., 2016; Wu et al., 2025), refers to a reduced capacity to experience pleasure or diminished responsiveness to pleasurable stimuli. In depression, anhedonia may involve a relatively stable trait component and has been found to be associated with a more persistent course of illness (Husain and Roiser, 2018; Lambert et al., 2018). Adolescence is a period of rapid development of the reward-processing system and, therefore, a crucial period for studying the relationship between anhedonia and depressive symptoms (Li P. et al., 2018). Accordingly, it is necessary to further explore the associated factors and statistical pathways that link anhedonia and depressive symptoms in adolescents.

As research on adolescent depression has advanced, depression has become widely regarded as closely linked to difficulties in emotion regulation (Zhao et al., 2017). Emotion regulation is the process by which individuals regulate their emotional responses by altering the intensity, duration, and expression of emotional experience (Gross, 1998). Emotion regulation capacity carries considerable adaptive significance for individual development and has important implications for cognitive functioning, the establishment of interpersonal relationships, and the maintenance of physical and mental health (Hao and Pan, 2020; Liu et al., 2020; Zhan et al., 2024). Emotion regulation encompasses not only intrapersonal but also interpersonal regulation in interactions. Interpersonal emotion regulation emphasizes that individuals regulate their emotional states through, or by influencing, others during social interactions (Dixon-Gordon et al., 2015; Hu et al., 2020; Niven, 2017; Reeck et al., 2016; Zaki and Williams, 2013). Recent studies further indicate that interpersonal emotion regulation has a distinct neurocognitive basis (Dong et al., 2024) and is closely associated with individuals' perceived social support, relationship quality, and mental health (Marroquín, 2011; Williams et al., 2018). From the perspective of early adolescent development, reward experiences, peer interactions, and support seeking are all undergoing rapid change. Higher levels of anhedonia may co-occur with reduced sharing of positive emotion, diminished social engagement, and less support seeking, and may therefore be linked to lower levels of interpersonal emotion regulation; lower levels of interpersonal emotion regulation may, in turn, co-occur with higher levels of depressive symptoms. Therefore, this study treats interpersonal emotion regulation as a mediator in the statistical association between anhedonia and depressive symptoms.

Adolescent interpersonal emotion regulation does not occur in isolation but is embedded within social contexts such as the family, peers, and school. Compared with peer support, the family remains an important arena for emotional expression, support seeking, and emotion socialization in early adolescence. According to the tripartite model of familial influence on children's emotion regulation, the family context may be linked to the development of emotion regulation through observational learning and modeling, parenting practices specific to emotion, and the emotional climate of the family (Morris et al., 2007). Family functioning describes the affective bonds, communication, role allocation, and rule operation among family members and constitutes an important family-system factor related to adolescent psychological adjustment (Fang et al., 2004; Li X. et al., 2018). Previous research has shown that higher family cohesion is associated with lower levels of depression (Bian et al., 2024), whereas poorer family functioning is associated with higher risk of negative psychological states such as depression and anxiety, as well as internalizing and externalizing problems in children and adolescents (Huang et al., 2022; Wang et al., 2022; Wen et al., 2024). Although early adolescents increasingly rely on peers, they still require the family to provide a sense of security, emotional modeling, and stable support. It can therefore be inferred that family functioning may alter the strength of the association between anhedonia and interpersonal emotion regulation; accordingly, this study specifies family functioning as a first-stage moderator.

In summary, the relationship between anhedonia and depressive symptoms is unlikely to follow a single pathway but may instead be jointly related to individuals' interpersonal emotion regulation resources and to the context of family functioning. Drawing on emotion regulation theory and the perspective of family emotion socialization, this study first tests a moderated mediation model in the full sample, using the total BDI-II-C score as a continuous dependent variable; subsequently, separate analyses of the below-cutoff and at-or-above-cutoff groups are conducted as exploratory sensitivity analyses to describe whether the model pathways remain consistent within the restricted score ranges produced by the cutoff-based grouping. Based on existing research, this study proposes the following hypotheses: (1) anhedonia is positively associated with depressive symptoms in adolescents; (2) interpersonal emotion regulation shows a significant indirect effect in the association between anhedonia and depressive symptoms; and (3) family functioning moderates the association between anhedonia and interpersonal emotion regulation. The conceptual model is presented in Figure 1.

**Figure 1:**
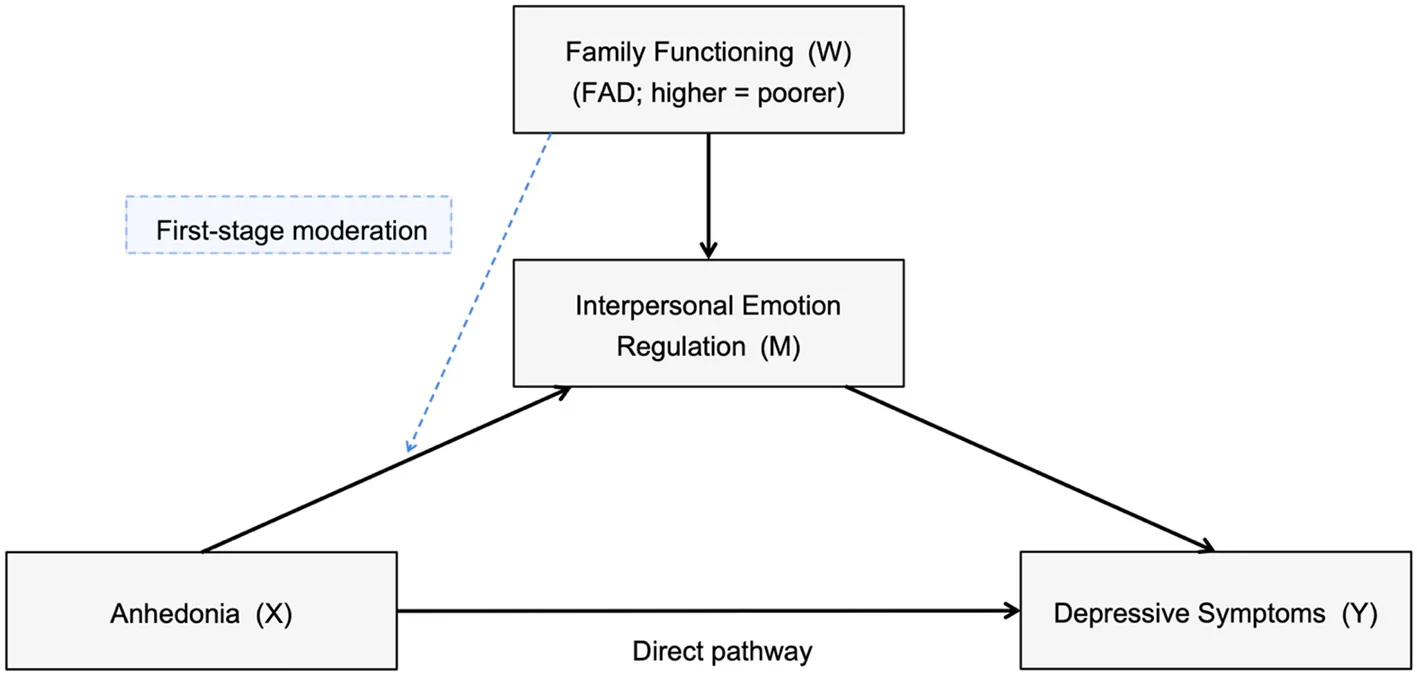
Conceptual model: the moderated mediation model. Higher SHAPS scores indicate more severe anhedonia; higher FAD scores indicate poorer family functioning. Family functioning moderates the first-stage path from anhedonia to interpersonal emotion regulation.

## 2 Materials and methods

### 2.1 Participants

This study used cluster sampling to recruit 1,635 students from two secondary schools in Hunan Province, including 540 in Grade 7, 551 in Grade 8, and 544 in Grade 9. Of these, 785 were boys (48%), and 850 were girls (52%), with a mean age of 12.73 years. The BDI-II-C is a screening measure of depressive symptoms rather than a clinical diagnostic instrument. Based on the BDI-II-C cutoff, 1,171 students were assigned to the below-cutoff group (BDI-II-C ≤ 13) and 464 to the at-or-above-cutoff group (BDI-II-C ≥ 14).

### 2.2 Research procedure

The study was approved by the Ethics Committee of Hengyang Normal University (approval number: 2026LLLW010). With the approval of the participating schools, the questionnaires were administered collectively on a class-by-class basis. Before the survey, students were informed of the purpose of the study, the anonymity of their responses, and the principle of voluntary participation, and informed consent was obtained from the students and their guardians. Students completed the questionnaires in a classroom setting under the supervision of researchers or schoolteachers who had received standardized instructions. The process took approximately 30 min. All questionnaire data were used solely for research analysis. Data were collected between January and March 2026.

### 2.3 Research instruments

#### 2.3.1 Beck Depression Inventory-II, Chinese version (BDI-II-C)

The BDI-II-C (Beck et al., 1996; Yang et al., 2014) assesses the severity of depressive symptoms over the past 2 weeks. It contains 21 items, each rated on a 4-point scale from 0 to 3, yielding total scores from 0 to 63. The scale is used for symptom screening, not for clinical diagnosis. The overall internal consistency coefficient in this study was 0.860. To assess potential overlap with the SHAPS regarding pleasure and interest, a 19-item depressive symptom score, excluding Items 4 (loss of pleasure) and 12 (loss of interest), was also constructed for robustness analyses.

#### 2.3.2 Snaith–Hamilton Pleasure Scale (SHAPS)

The SHAPS (Snaith et al., 1995) consists of 14 items assessing hedonic experience across the domains of interests/pastimes, social interactions, sensory experience, and food/drink. Each item is rated on a 4-point scale. In the version administered in this study, responses were coded from 1 (strongly disagree) to 4 (strongly agree), with higher raw scores reflecting greater hedonic capacity. To align the scoring direction with the severity of anhedonia, all items were reverse-scored, so that the total score (range 14–56) followed the continuous scoring method of Franken et al. (2007), whereby higher scores indicate more severe anhedonia. Continuous scoring, rather than the original dichotomous scoring (Snaith et al., 1995; range 0–14), was used because it retains more variance and is better suited to correlation and regression analyses in a non-clinical sample, whereas the dichotomous score was designed mainly for clinical screening. All statistical analyses reported in this article are based on the reverse-scored SHAPS scores. The overall internal consistency coefficient in this study was 0.888.

#### 2.3.3 Interpersonal Emotion Regulation Questionnaire (IERQ)

The IERQ (Hofmann et al., 2016) comprises 20 items across four dimensions: enhancing positive affect, perspective-taking, soothing, and social modeling. Items are rated on a 5-point scale, with higher scores indicating greater interpersonal emotion regulation. The overall internal consistency coefficient in this study was 0.904.

#### 2.3.4 Chinese version of the Family Assessment Device (FAD)

This scale was revised by Liu and He (1991) based on the McMaster Model of Family Functioning (Epstein et al., 1983). It includes 60 items across seven dimensions: problem solving, communication, roles, affective responsiveness, affective involvement, behavior control, and general functioning. In this study, the raw FAD total score was reported in the correlation analyses, whereas the FAD total score was standardized in the regression and PROCESS models. When interpreting the results, low FAD scores indicate healthier family functioning, and high FAD scores indicate poorer family functioning. The overall internal consistency coefficient in this study was 0.891.

### 2.4 Data processing and statistical analysis

SPSS and the PROCESS macro were used for statistical analysis. First, descriptive statistics and correlation analyses were conducted, and Welch's independent samples *t-tests*, which do not assume equal variances, were used to examine gender differences, and one-way analysis of variance (ANOVA) with Tukey's *post hoc* tests was used to examine grade differences. Second, in the full sample, with the total BDI-II-C score as a continuous dependent variable, PROCESS Model 4 was used to test the indirect effect, and PROCESS Model 7, following Wen and Ye (2014), was used to test the moderating role of family functioning in the pathway linking anhedonia to interpersonal emotion regulation, with 5,000 bootstrap resamples and gender and grade as covariates. Third, the full-sample models were repeated after removing Items 4 and 12 from the BDI-II-C total score to examine the robustness of the results to overlap in item content. Finally, exploratory subgroup sensitivity analyses were conducted using the BDI-II-C cutoff. Given that cross-sectional data cannot establish temporal ordering, the mediation and moderation results reported in this study are interpreted as statistical associations, and no causal inferences are drawn.

## 3 Results

### 3.1 Common method bias test

The Harman single-factor test was used to examine common method bias. All 115 items were entered into an unrotated exploratory factor analysis. The results showed that 26 factors had eigenvalues greater than 1, and the first factor accounted for 11.45% of the variance, below the 40% critical threshold, suggesting that no single factor accounted for the majority of the covariance. However, because Harman's test is a relatively insensitive diagnostic (Podsakoff et al., 2003), this result does not rule out common method variance; this issue is addressed further in the Limitations.

### 3.2 Descriptive statistics and correlation analysis of variables

Welch's independent samples *t-tests* showed significant gender differences in depressive symptoms, anhedonia, and raw FAD total scores: depressive symptoms (*t* = −2.097, *p* = 0.036, Cohen's *d* = −0.104); anhedonia (*t* = 2.377, *p* = 0.018, Cohen's *d* = 0.118); and family functioning (*t* = −5.400, *p* < 0.001, Cohen's *d* = −0.268). Gender differences in interpersonal emotion regulation were not significant (*t* = 1.382, *p* = 0.167, Cohen's *d* = 0.069). Descriptive statistics indicated that girls reported higher levels of depressive symptoms and comparatively poorer family functioning, whereas boys reported slightly higher anhedonia scores.

One-way ANOVA showed a significant difference in depressive symptoms across grades (*F* = 8.214, *p* < 0.001). Tukey's *post hoc* tests indicated that students in Grades 8 and 9 scored significantly higher on depressive symptoms than those in Grade 7, whereas the difference between Grades 8 and 9 was not significant. No significant grade differences were found in anhedonia, interpersonal emotion regulation, or raw FAD total scores (*F* = 1.110, *p* = 0.330; *F* = 2.067, *p* = 0.127; *F* = 0.071, *p* = 0.932, respectively). Descriptive statistics for all variables by gender and grade are presented in Table 1.

**Table 1:** Descriptive statistics of variables by gender and grade (*M* ± *SD*).

Variable	Gender		Grade		
	Boys (*n* = 785)	Girls (*n* = 850)	Grade 7 (*n* = 540)	Grade 8 (*n* = 551)	Grade 9 (*n* = 544)
Depressive Symptoms	9.98 ± 8.15	10.85 ± 8.57	9.33 ± 8.75	11.36 ± 8.48	10.58 ± 7.78
Anhedonia	24.38 ± 7.37	23.53 ± 7.11	24.17 ± 7.33	23.56 ± 7.20	24.08 ± 7.21
Interpersonal Emotion Regulation	62.88 ± 17.75	61.71 ± 16.17	61.08 ± 17.84	63.05 ± 16.32	62.67 ± 16.65
Family Functioning	118.46 ± 24.43	124.77 ± 22.68	121.91 ± 24.16	121.88 ± 22.49	121.43 ± 24.58

Values are raw scores. Higher SHAPS scores indicate more severe anhedonia; higher FAD scores indicate poorer family functioning.

An ANOVA with depressive symptoms scores as the dependent variable and gender and grade as independent variables showed no significant interaction between gender and grade (*F* = 0.856, *p* = 0.425). Correlation analysis showed that family functioning was not significantly correlated with depressive symptoms (*r* = 0.019, *p* = 0.452); anhedonia was significantly positively correlated with depressive symptoms (*r* = 0.329, *p* < 0.001); interpersonal emotion regulation was significantly negatively correlated with depressive symptoms (*r* = −0.319, *p* < 0.001); and anhedonia was significantly negatively correlated with interpersonal emotion regulation (*r* = −0.454, *p* < 0.001). Family functioning was significantly positively correlated with anhedonia (*r* = 0.194, *p* < 0.001) and significantly negatively correlated with interpersonal emotion regulation (*r* = −0.213, *p* < 0.001), indicating that poorer family functioning was associated with higher anhedonia and lower interpersonal emotion regulation. Table 2 presents the descriptive statistics and correlation matrix for all variables.

**Table 2:** Descriptive statistics and correlation matrix.

Variable	*M*	*SD*	1	2	3
1. Depressive symptoms	10.43	8.38			
2. Family functioning	121.74	23.74	0.019		
3. Anhedonia	23.94	7.25	0.329^***^	0.194^***^	
4. Interpersonal emotion regulation	62.27	16.95	−0.319^***^	−0.213^***^	−0.454^***^

Values are raw scores. Higher SHAPS scores indicate more severe anhedonia; higher FAD scores indicate poorer family functioning. ^***^p < 0.001.

### 3.3 Moderated mediation model in the full sample

In the full sample, with the BDI-II-C total score as a continuous dependent variable and gender and grade as covariates, PROCESS Model 4 was first used to test the indirect effect of interpersonal emotion regulation. The total association between anhedonia and depressive symptoms was significant, β = 0.335, *SE* = 0.023, *t* = 14.298, *p* < 0.001, 95% CI [0.289, 0.380]. Anhedonia was significantly negatively associated with interpersonal emotion regulation (β = −0.458, *SE* = 0.022, *t* = −20.777, *p* < 0.001, 95% CI [−0.501, −0.414]). When anhedonia and interpersonal emotion regulation were entered simultaneously, interpersonal emotion regulation was significantly negatively associated with depressive symptoms (β = −0.213, *SE* = 0.026, *t* = −8.264, *p* < 0.001, 95% CI [−0.264, −0.163]), and the direct association between anhedonia and depressive symptoms remained significant (β = 0.237, *SE* = 0.026, *t* = 9.193, *p* < 0.001, 95% CI [0.187, 0.288]). Bootstrap testing showed an indirect effect of 0.097, 95% CI [0.072, 0.123].

PROCESS Model 7 was used to test the moderating role of family functioning in the first-stage path. The complete regression results are presented in Table 3, and the standardized paths are shown in Figure 2. In the model including family functioning and the interaction term, anhedonia was significantly negatively associated with interpersonal emotion regulation (β = −0.418, *SE* = 0.022, *t* = −18.599, *p* < 0.001, 95% CI [−0.462, −0.374]). Family functioning was significantly negatively associated with interpersonal emotion regulation (β = −0.127, *SE* = 0.022, *t* = −5.644, *p* < 0.001), and the interaction term between anhedonia and family functioning was significant (β = 0.089, *SE* = 0.021, *t* = 4.327, *p* < 0.001, 95% CI [0.049, 0.129]). At low, medium, and high FAD scores, the conditional indirect effects were 0.107, 0.088, and 0.069, respectively, and none of the bootstrap 95% CIs included zero (see Table 4). The index of moderated mediation was −0.019, 95% CI [−0.030, −0.009]. Because higher FAD scores indicate poorer family functioning, these results indicate that the conditional indirect effect linking anhedonia to depressive symptoms through interpersonal emotion regulation is stronger when family functioning is healthier.

**Table 3:** Regression results for the moderated mediation model in the full sample.

Dependent variable/Predictor	*R^2^*	β	*SE*	*t*	*p*	95% CI
Interpersonal emotion regulation as the dependent variable	0.234					
Constant		−0.255	0.222	−1.150	0.250	[−0.691, 0.180]
Anhedonia		−0.418	0.022	−18.599	< 0.001	[−0.462, −0.374]
Family functioning		−0.127	0.022	−5.644	< 0.001	[−0.171, −0.083]
Anhedonia × family functioning		0.089	0.021	4.327	< 0.001	[0.049, 0.129]
Gender		−0.084	0.044	−1.919	0.055	[−0.170, 0.002]
Grade		0.046	0.027	1.714	0.087	[−0.007, 0.098]
Depressive symptoms as the dependent variable	0.152					
Constant		−0.839	0.234	−3.582	< 0.001	[−1.299, −0.380]
Anhedonia		0.237	0.026	9.193	< 0.001	[0.187, 0.288]
Interpersonal emotion regulation		−0.213	0.026	−8.264	< 0.001	[−0.264, −0.163]
Gender		0.112	0.046	2.448	0.014	[0.022, 0.203]
Grade		0.084	0.028	2.984	0.003	[0.029, 0.139]

All continuous variables were standardized before analysis, and all coefficients are standardized. Higher SHAPS scores indicate more severe anhedonia; higher FAD scores indicate poorer family functioning.

**Figure 2:**
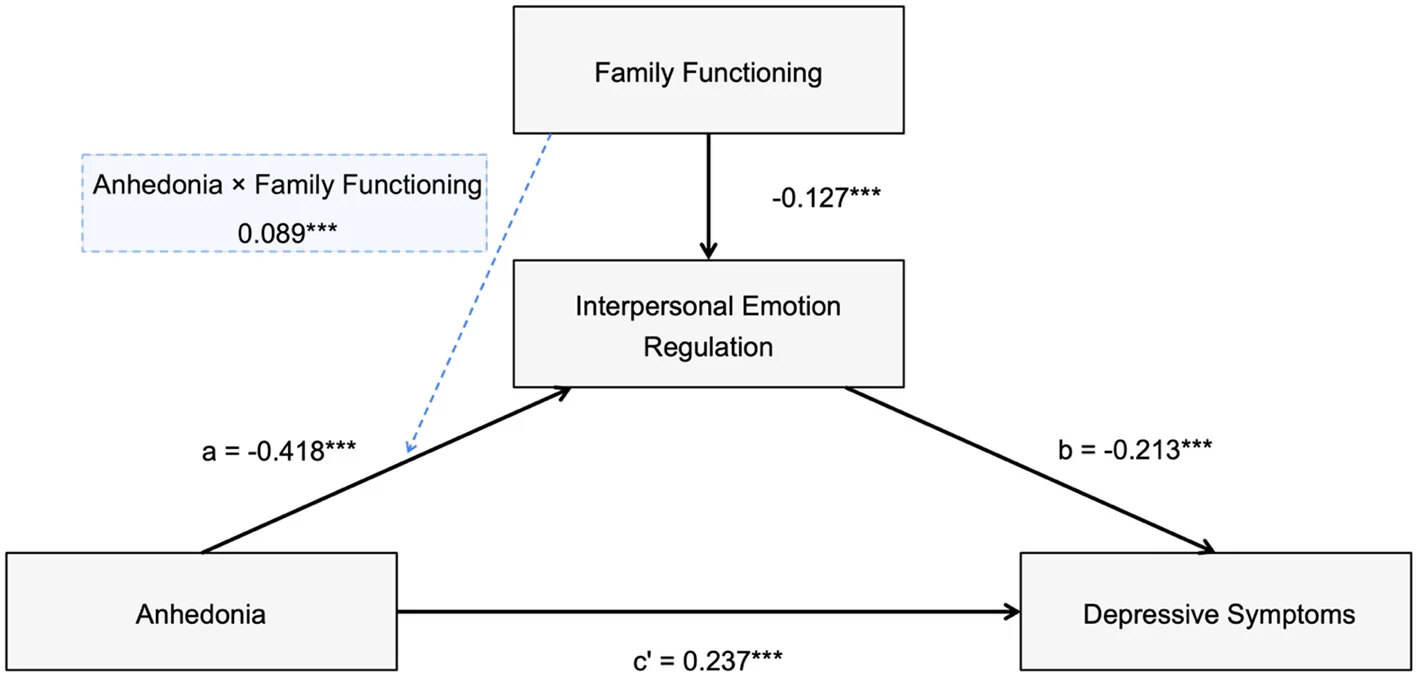
Standardized moderated mediation model in the full sample. All coefficients are standardized. Higher SHAPS scores indicate more severe anhedonia; higher FAD scores indicate poorer family functioning. ***p < 0.001.

**Table 4:** Conditional indirect effects and index of moderated mediation in the full sample.

Level of family functioning	Standardized value	Effect	Bootstrap *SE*	95% CI
Low FAD	−1 *SD*	0.107	0.015	[0.079, 0.136]
Medium FAD	*M*	0.088	0.012	[0.066, 0.112]
High FAD	+1 *SD*	0.069	0.011	[0.049, 0.093]
Index of moderated mediation		−0.019	0.005	[−0.030, −0.009]

Low, medium, and high FAD refer to −1 SD, the mean, and +1 SD of the standardized FAD score. Higher FAD scores indicate poorer family functioning.

### 3.4 Item-overlap robustness analysis and subgroup sensitivity analysis

Items 4 and 12 were removed from the BDI-II-C total score to construct a 19-item depressive symptoms score, and the full-sample analyses were repeated. The total association between anhedonia and the revised depressive symptoms score remained significant (β = 0.318, *SE* = 0.023, *t* = 13.521, *p* < 0.001, 95% CI [0.272, 0.364]). When interpersonal emotion regulation was included, the direct association between anhedonia and the revised depressive symptoms score remained significant (β = 0.223, *SE* = 0.026, *t* = 8.597, *p* < 0.001, 95% CI [0.172, 0.274]), and interpersonal emotion regulation was significantly negatively associated with the revised depressive symptoms score (β = −0.207, *SE* = 0.026, *t* = −7.991, *p* < 0.001, 95% CI [−0.258, −0.156]). The bootstrap test showed an indirect effect of 0.095, 95% CI [0.071, 0.121], and an index of moderated mediation of −0.018, 95% CI [−0.029, −0.009]. Because the robustness analysis adjusted only the scoring of the depressive symptoms outcome, the coefficients of the first-stage moderation model remained unchanged; the index of moderated mediation nevertheless differed slightly, as it also incorporates the path from interpersonal emotion regulation to the outcome. Compared with the original 21-item BDI-II-C model, the direction and significance of all paths were consistent, indicating that the main results are reasonably robust to overlap in scale content; a detailed comparison is presented in Table 5.

**Table 5:** Robustness analysis after removing overlapping BDI-II-C items.

Index	Original BDI-II-C	95% CI	After Removing Items 4 and 12	95% CI
Total association between anhedonia and depressive symptoms (path *c*)	0.335	[0.289, 0.380]	0.318	[0.272, 0.364]
Direct association between anhedonia and depressive symptoms (path *c′*)	0.237	[0.187, 0.288]	0.223	[0.172, 0.274]
Association between interpersonal emotion regulation and depressive symptoms (path *b*)	−0.213	[−0.264, −0.163]	−0.207	[−0.258, −0.156]
Indirect effect (*ab*)	0.097	[0.072, 0.123]	0.095	[0.071, 0.121]
Index of moderated mediation	−0.019	[−0.030, −0.009]	−0.018	[−0.029, −0.009]

Exploratory subgroup sensitivity analyses were then conducted using the BDI-II-C cutoff. The complete regression results for the below-cutoff group are presented in Table 6. The standard deviation of BDI-II-C total scores in the full sample was 8.383; after grouping by the cutoff, the standard deviations were 3.849 in the below-cutoff group and 6.774 in the at-or-above-cutoff group. Within-group dispersion in both subgroups was lower than in the full sample, indicating range restriction of the dependent variable following cutoff-based grouping.

**Table 6:** Test of moderated mediation effect in the below-cutoff group.

Dependent variable/Predictor	*R^2^*	β	*SE*	*t*	*p*	95% CI
Interpersonal emotion regulation as the dependent variable	0.208					
Constant		0.078	0.249	0.310	0.753	[−0.410, 0.567]
Gender		−0.062	0.050	−1.240	0.217	[−0.161, 0.037]
Grade		0.013	0.030	0.420	0.675	[−0.046, 0.072]
Anhedonia		−0.398	0.029	−13.670	< 0.001	[−0.455, −0.341]
Family functioning		−0.130	0.027	−4.850	< 0.001	[−0.182, −0.077]
Anhedonia × family functioning		0.097	0.026	3.670	< 0.001	[0.045, 0.149]
Depressive symptoms as the dependent variable	0.054					
Constant		−1.032	0.132	−7.840	< 0.001	[−1.291, −0.774]
Gender		0.034	0.026	1.280	0.201	[−0.018, 0.086]
Grade		0.062	0.016	3.870	< 0.001	[0.030, 0.093]
Interpersonal emotion regulation		−0.061	0.015	−4.020	< 0.001	[−0.091, −0.031]
Anhedonia		0.061	0.016	3.750	< 0.001	[0.029, 0.093]

All continuous variables were standardized before analysis, and all coefficients are standardized. Higher SHAPS scores indicate more severe anhedonia; higher FAD scores indicate poorer family functioning.

In the below-cutoff group, the conditional indirect effects were significant at low, medium, and high levels of FAD scores, with effect values of 0.030, 0.024, and 0.018, respectively, and none of the 95% CIs included zero. The index of moderated mediation was −0.006, 95% CI [−0.012, −0.002] (see Table 7). Because lower FAD scores indicate healthier family functioning, the conditional indirect effect was stronger at low FAD scores (see Figure 3). These subgroup results are reported only as exploratory sensitivity analyses and are not taken as evidence that different psychological mechanisms operate at different levels of depressive symptoms.

**Table 7:** Conditional indirect effects at different levels of family functioning in the below-cutoff group.

Level of family functioning	Standardized value	Effect	Lower 95% CI	Upper 95% CI
Low FAD	−0.970	0.030	0.015	0.047
Medium FAD	0.010	0.024	0.012	0.037
High FAD	0.980	0.018	0.009	0.029
Index of moderated mediation		−0.006	−0.012	−0.002

Values are the subgroup mean ± 1 SD of the FAD score standardized in the full sample, and therefore differ slightly from −1, 0, and +1.

**Figure 3:**
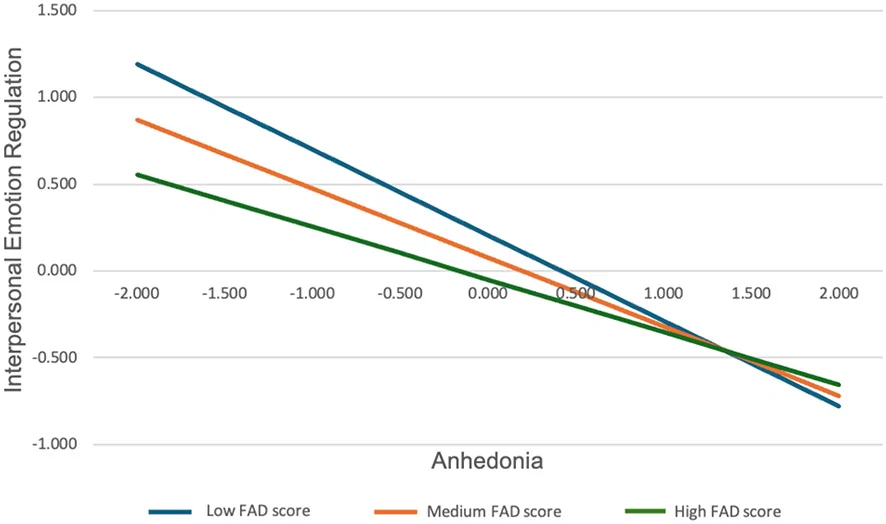
Simple-slopes plot of the moderating effect of family functioning on the relationship between anhedonia and interpersonal emotion regulation in the below-cutoff group. Higher SHAPS scores indicate more severe anhedonia. Low, medium, and high in the legend refer to levels of the FAD score; lower FAD scores indicate healthier family functioning, whereas higher FAD scores indicate poorer family functioning.

To examine the stability of the model pathways within a restricted score range, exploratory sensitivity analyses were further conducted for the at-or-above-cutoff group (BDI-II-C total score of 14–63). The results are presented in Table 8 and Figure 4.

**Figure 4:**
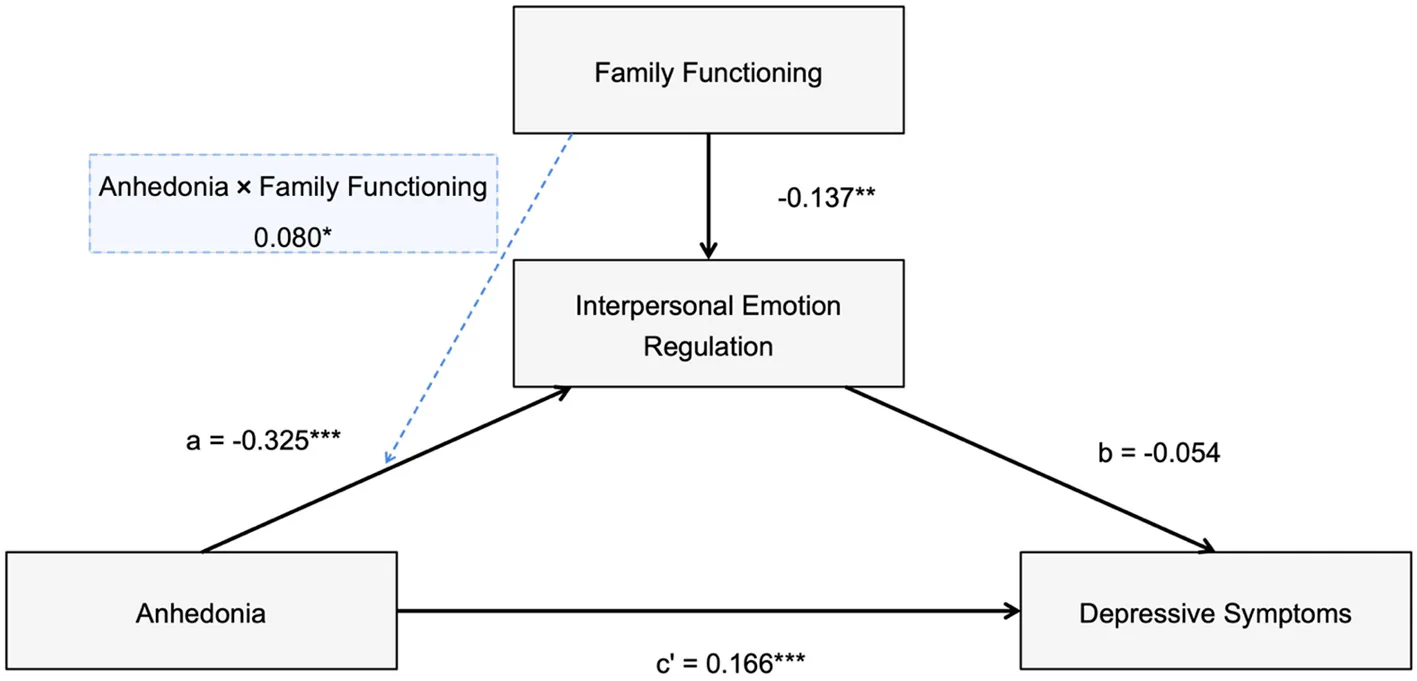
Standardized moderated mediation model in the at-or-above-cutoff group. All coefficients are standardized. Higher SHAPS scores indicate more severe anhedonia; higher FAD scores indicate poorer family functioning. *p < 0.05; **p < 0.01; ***p < 0.001.

**Table 8:** Test of the moderated mediation effect in the at-or-above-cutoff group.

Dependent variable/Predictor	*R^2^*	β	*SE*	*t*	*p*	95% CI
Interpersonal emotion regulation as the dependent variable	0.206					
Constant		−1.661	0.447	−3.710	< 0.001	[−2.539, −0.782]
Gender		−0.070	0.084	−0.830	0.407	[−0.236, 0.096]
Grade		0.180	0.052	3.440	< 0.001	[0.077, 0.283]
Anhedonia		−0.325	0.037	−8.710	< 0.001	[−0.399, −0.252]
Family functioning		−0.137	0.043	−3.170	0.002	[−0.222, −0.052]
Anhedonia × family functioning		0.080	0.034	2.360	0.019	[0.013, 0.146]
Depressive symptoms as the dependent variable	0.081					
Constant		1.763	0.400	4.410	< 0.001	[0.977, 2.549]
Gender		0.089	0.073	1.220	0.223	[−0.054, 0.233]
Grade		−0.086	0.047	−1.830	0.068	[−0.178, 0.006]
Interpersonal emotion regulation		−0.054	0.041	−1.310	0.189	[−0.134, 0.027]
Anhedonia		0.166	0.035	4.690	< 0.001	[0.096, 0.235]

All continuous variables were standardized before analysis, and all coefficients are standardized. Higher SHAPS scores indicate more severe anhedonia; higher FAD scores indicate poorer family functioning.

As shown in Table 8, in the at-or-above-cutoff group, anhedonia was significantly negatively associated with interpersonal emotion regulation (β = −0.325, *SE* = 0.037, *t* = −8.710, *p* < 0.001, 95% CI [−0.399, −0.252]), family functioning was likewise significantly negatively associated with interpersonal emotion regulation (β = −0.137, *SE* = 0.043, *t* = −3.170, *p* = 0.002), and the interaction term between anhedonia and family functioning was significant (β = 0.080, *SE* = 0.034, *t* = 2.360, *p* = 0.019). However, the regression association between interpersonal emotion regulation and depressive symptoms did not reach significance (β = −0.054, *SE* = 0.041, *t* = −1.310, *p* = 0.189). Because this group was defined by the cutoff of the same dependent variable, the attainable range of BDI-II-C scores was restricted. The non-significant interpersonal emotion regulation path may therefore be related to range restriction, and may also be affected by sample size, within-group distribution, and other uncontrolled factors; it cannot be taken as evidence that a different psychological mechanism operates in this group. The conditional indirect effects are presented in Table 9: at low, medium, and high FAD scores, the conditional indirect effects were 0.021, 0.017, and 0.013, respectively, with corresponding 95% CIs that all included zero. The index of moderated mediation was −0.004, 95% CI [−0.013, 0.002].

**Table 9:** Conditional indirect effects at different levels of family functioning in the at-or-above-cutoff group.

Level of family functioning	Standardized value	Effect	Lower 95% CI	Upper 95% CI
Low FAD	−1 *SD*	0.021	−0.010	0.054
Medium FAD	*M*	0.017	−0.008	0.043
High FAD	+1 *SD*	0.013	−0.006	0.035
Index of moderated mediation		−0.004	−0.013	0.002

Low, medium, and high FAD refer to −1 SD, the mean, and +1 SD of the standardized FAD score. Higher FAD scores indicate poorer family functioning.

## 4 Discussion

Focusing on the relationship between anhedonia and depressive symptoms in adolescents, this study examined a moderated mediation model at the levels of interpersonal emotion regulation and family functioning. The results showed that anhedonia was significantly positively associated with depressive symptoms in the full sample. Interpersonal emotion regulation partially mediated this association, and family functioning further moderated the association between anhedonia and interpersonal emotion regulation. The results remained stable after the two BDI-II-C items overlapping in content with pleasure and interest were removed. It should be emphasized that this study used a cross-sectional questionnaire design, and that all results reflect concurrent statistical associations among the variables; neither temporal ordering nor causal direction can be established.

### 4.1 The statistical role of interpersonal emotion regulation in the relationship between anhedonia and depressive symptoms in adolescents

The results showed that anhedonia was significantly and positively associated with depressive symptoms in adolescents. The full-sample model further indicated that interpersonal emotion regulation partially mediated this association. This finding suggests that higher levels of anhedonia may co-occur with less sharing of positive emotion, less support seeking, and less perspective taking, and may, in turn, be linked to higher levels of depressive symptoms. The robustness analysis conducted after removing Items 4 and 12 of the BDI-II-C yielded comparable direct and indirect effects and indices of moderated mediation, which reduces the threat that overlap in scale content poses to the interpretation of the main conclusions. The exploratory subgroup analyses showed that the path from interpersonal emotion regulation to depressive symptoms did not reach significance in the at-or-above-cutoff group; this result may, however, be affected by the range restriction produced by grouping on the same dependent variable, and a non-significant path within this subgroup alone cannot be taken to indicate that a different psychological mechanism operates in this group.

It should be further noted that the negative association between anhedonia and interpersonal emotion regulation was significant in the full sample and in both subgroups. Although the coefficient was smaller in the at-or-above-cutoff group, the direction and significance of this path were consistent across all three analyses, indicating that the first segment of the model remained stable when the range of the dependent variable was restricted. A possible interpretation is that adolescents with higher levels of anhedonia derive less pleasure from social activities and interpersonal contact; the interpersonal behaviors assessed by the IERQ may therefore become less rewarding, and reduced responsiveness to social reward may be accompanied by less frequent use of interpersonal resources for regulating negative affect. This interpretation describes a concurrent association rather than a temporal sequence.

### 4.2 The moderating role of family functioning in the relationship between anhedonia and interpersonal emotion regulation

The study also found that family functioning moderated the concurrent association between anhedonia and interpersonal emotion regulation. Because lower FAD scores indicate healthier family functioning, the results should be interpreted as follows: when family functioning is healthier, the conditional indirect effect linking anhedonia to depressive symptoms through interpersonal emotion regulation is stronger, whereas when family functioning is poorer, this conditional indirect effect is relatively weaker. This result does not imply that family functioning directly causes changes in depressive symptoms; rather, it indicates that family affective bonds, communication quality, and a supportive environment may constitute an important context for adolescents' interpersonal emotion regulation.

In addition to its moderating role, family functioning was significantly negatively associated with interpersonal emotion regulation in the full sample and in both subgroups. Because higher FAD scores indicate poorer family functioning, this negative coefficient means that adolescents from families with poorer functioning reported lower levels of interpersonal emotion regulation. This finding also clarifies the position of family functioning in the present model: in this sample, family functioning was significantly associated with anhedonia and with interpersonal emotion regulation but not with depressive symptoms, which is consistent with modeling it as a first-stage moderator rather than as a direct correlate of the outcome.

The moderating role of family functioning can be understood from the perspectives of motivation and parent–child communication. First, the effectiveness of interpersonal emotion regulation depends, to some extent, on adolescents' perceptions of interpersonal interactions. Research has found that certain types of parents (e.g., authoritarian parents) may undermine adolescents' intrinsic motivation for interpersonal interaction, and that such adolescents are more likely to display emotions such as anger and depression and to show externalizing behavior problems (Ebrahimi et al., 2017; Sheng et al., 2019). A highly controlling family climate tends to generate higher interpersonal-interaction stress and negative experiences such as depression and anxiety in adolescents, thereby weakening their willingness to proactively engage in interpersonal emotion regulation (Cui et al., 2014; Lin and Lin, 2024; Zhu et al., 2024). Second, family functioning reflects the interaction patterns, intimacy levels, and overall climate among family members. In families with poorer functioning, reduced parent–child interaction, lower intimacy, and stressors such as parental conflict increase adolescents' emotional insecurity and risk of depression (Davies and Cummings, 1994; Wang et al., 2018, 2020). Poor family functioning is also an important risk factor for adolescents' problem behaviors and internalizing issues (Wang et al., 2022; Wen et al., 2024). Conversely, families with better functioning generally provide more stable emotional support and communication opportunities, helping adolescents learn to express emotions, seek support, and regulate relationships in family and peer interactions. The processes of interpersonal emotion regulation between parents and adolescents may also be closely associated with adolescents' depressive symptoms (Gadassi-Polack et al., 2025). Therefore, family functioning helps to specify the conditions under which the association between anhedonia and interpersonal emotion regulation varies.

### 4.3 Implications and limitations

This study further examined the concurrent statistical associations among anhedonia, interpersonal emotion regulation, family functioning, and depressive symptoms in adolescents. The results identify factors related to adolescent depressive symptoms that may serve as a reference for future work on this topic: (1) anhedonia was stably and positively associated with depressive symptoms; (2) interpersonal emotion regulation, including enhancing positive affect, perspective taking, soothing, and social modeling, partially accounted for the association between anhedonia and depressive symptoms; and (3) family functioning was associated with both anhedonia and interpersonal emotion regulation, and it moderated the first-stage path. Within the limits of a cross-sectional design, these associations suggest that adolescents' interpersonal support and help-seeking, and the family context in which these develop, are worth attention in school mental health work. Researchers interested in this topic may consider these variables as candidates for longitudinal or experimental studies.

This study has the following limitations. First, the specific mechanisms underlying the association between family functioning and interpersonal emotion regulation remain to be further verified by subsequent longitudinal or experimental research, because this study used a cross-sectional design and cannot establish the temporal order or causal direction among the variables. Second, all variables were measured entirely through adolescents' self-reports collected in a single session. Although Harman's single-factor test did not indicate that one factor dominated the data, this test has limited sensitivity, and more robust procedures, such as an unmeasured latent method construct or a marker variable, were not applied. Accordingly, common method variance and social desirability may have inflated the observed associations. Future research should incorporate multiple informants (e.g., parent reports of family functioning), temporally separated measurement, and statistical controls for method variance. Third, the existing data did not capture demographic variables such as family socioeconomic status, urban vs. rural residence, only-child status, and family structure, so the covariates included in the models remain limited. Future research should include these variables to reduce omitted-variable bias. Fourth, dividing subgroups according to the BDI-II-C cutoff narrows the attainable range of the dependent variable within each group and may affect regression coefficients and their significance; the subgroup results were therefore treated only as exploratory sensitivity analyses, and no substantive inferences were drawn about differences in psychological mechanisms between groups. Fifth, the study reported reliability mainly at the level of total scale scores; future research should further report the reliability of each subscale and use confirmatory factor analysis to test structural validity.

## 5 Conclusion

(1) Anhedonia is significantly positively associated with depressive symptoms in adolescents.

(2) Interpersonal emotion regulation partially mediates the association between anhedonia and depressive symptoms in adolescents; exploratory subgroup analyses showed that this mediating effect was significant in the below-cutoff group but not in the at-or-above-cutoff group, a difference that may reflect range restriction rather than a difference in mechanism. After Items 4 and 12 of the BDI-II-C were removed, the main paths, indirect effect, and index of moderated mediation in the full sample remained stable.

(3) Family functioning moderates the association between anhedonia and interpersonal emotion regulation; when family functioning is healthier, the conditional indirect effect linking anhedonia to depressive symptoms through interpersonal emotion regulation is stronger. The subgroup results based on the BDI-II-C cutoff are reported only as exploratory sensitivity analyses.

## Data Availability

The datasets presented in this article are not readily available because they contain sensitive information about minors, and public data sharing was not covered by the informed consent obtained from participants and their guardians. De-identified data are available from the corresponding authors upon reasonable request, subject to approval by the Ethics Committee of Hengyang Normal University. Requests to access the datasets should be directed to Siying Lin, lsyzyl0219@163.com; Fanggui Tang, tfg1618@126.com.
